# Lectures

**Published:** 1844-07-27

**Authors:** Benjamin Collins Brodie

**Affiliations:** Consulting-Surgeon of the Hospital


### LECTURES

*Delivered in the Theatre of St. George's Hospital, in the session* 1843–44,

BY SIR BENJAMIN COLLINS BRODIE,

Consulting-Surgeon of the Hospital.

### EXTRACTION OF FOREIGN BODIES.

*Foreign bodies in the nasal passages—In the external ear—In the digestive tube. Removal of fish-bones from the throat. Impaction of bodies in the œsophagus. Foreign bodies in the stomach. Knife swallowing, and injurious bodies in the intestines. General treatment.*

Gentlemen,—Two or three years ago I was consulted concerning a young person, a female, who had some complaint in her nostrils. There was a putrid discharge from them, and those symptoms were present which usually indicate the presence of diseased or dead bone of the nostrils; and presuming that this was the nature of the case I prescribed sarsaparilla, and treated her accordingly. This complaint had been going on since she was quite a child, and when I saw her she was eleven or twelve years of age. Not long ago, in blowing her nose, something came out of her nostrils—a large solid substance. Her family thought that this was the piece of dead bone which was expected to appear, and it was sent to me; but, on examining it, I found that it was not bone, nor had it the appearance of ever having been organised. It was convex on one side and concave on the other, and seemed to have been formed upon a nucleus. Dr. Prout was good enough to examine it chemically, and he found it to consist of dry mucus, with phosphate of lime, such as is secreted by an inflamed mucous membrane. The mucous membrane of the nose, like that of the bladder, will, when irritated, secrete phosphate of lime. I was led, from this, to conclude that, originally, some foreign substance had been introduced into the nose, and if it were a round body this would account for the concavity on one side of the concretion. Here was a case in which there was great reason to believe that some foreign body had been introduced into the nostrils, and had remained there for years, producing all the symptoms usually arising from diseased bone.

A little boy was brought to me a few years ago, with a putrid discharge from the nostrils. There, also, I thought that there was a piece of diseased bone. He had this for one or two years. On looking into the nostril, however, I perceived, at the upper part, something rather larger than a piece of dead bone might be supposed to present. I took hold of it with the forceps, and on removing it, found it was a tamarind-stone which the boy had thrust into the nostrils a year or two before, no one knowing anything of it. In each of these, when the foreign body was taken away, the symptoms subsided.

Another patient was brought to me supposed to have diseased bone in the nose,—a little girl in whom there had been a putrid discharge for two or three years. There I could see nothing, but, from the symptoms, I concluded that disease was going on in the bone. I prescribed for this patient sarsaparilla, and one morning something was blown out of her nose. It was brought to me, and I discoverd that it was a piece of sponge that had stuck in the nostril, and was now filled with mucus, and, I suppose, some phosphate of lime. As no one knew the history of the case I suppose that the child must have thrust it in herself. It is not very uncommon for children to get foreign bodies into their nostrils, and these cases show that you may be led into great error by supposing that there is diseased bone, when there is none at all.

In two of these cases the foreign body was blown out—came away spontaneously; and in the case of the tamarind-stone I removed it very easily with a pair of forceps. Other means, however, may be adopted for removing these foreign bodies. A child was brought to me who had got a glass bead into the nostril, and it was known that it was there. I tried to take hold of it with the forceps but they slipped over its smooth surface. I then introduced a probe, bent in a peculiar manner, which, getting behind the bead, pulled it out.

Foreign bodies may get into the external meatus of the ear. A child was brought to me who had got a broken piece of slate-pencil about half an inch in length, in the meatus. You might suppose it an easy matter to get a foreign body out of the external meatus of the ear, that part being so much more in sight than the nostril. But it is often very difficult, and for this reason: in the nose you may poke with the forceps, and do no harm. I have already stated what great manipulations the nostril will bear. But what will happen if you poke with the forceps in the ear? A child was brought to this hospital with a pea in the ear. A great many attempts had been made to remove it prior to the child being brought here. The pea was then out of sight, and the child had very alarming symptoms of inflammation of the brain. The little patient died; and it was found that in attempting to extract the pea, the membrana tympani had been destroyed. The injudicious poking of the tympanum with the forceps had caused inflammation of the bone of the tympanum, and a separation between it and the dura mater, so that the child died in consequence of the rude introduction of the forceps into the ear. Indeed, it is a very difficult thing to extract a foreign body from the ear with forceps, and if you attempt it you must proceed with the greatest caution. I have, however, extricated foreign bodies from the ear with a narrow pair of forceps by letting the rays of the sun shine into the meatus and then introducing the forceps, so that one blade came upon each side of the foreign body. But if you attempt it without the rays of the sun shining into the ear, and using your eyes carefully, and your hands slowly and attentively, nothing is more easy than to drive the body against the membrana tympani, break the latter, and push the body into the tympanum. I do not say that you are not to extract foreign bodies from the ear with forceps, but you must do it with the greatest care; for the want of care may lead to the destruction of the patient. But I have more frequently succeeded in these cases by other means. I stated that a child was brought to me with a piece of slate-pencil ih the ear. I placed the child opposite the light, and injected some tepid water into the ear with a syringe. There was room for the water to penetrate into the meatus, and as it came back it washed out the slate-pencil. There was a case brought into the hospital in which there was some foreign body—I believe a pea—in the external meatus. I tried all sorts of methods to get it out. I could not use the forceps, and it nearly filled up the meatus, so that either water could not pass behind it, or it was so jammed that the water injected by the syringe would not wash it out. I said, "Let it alone, let it remain there, the pea in all probability will dry and waste of itself, and then it will come out, or when it is rotten it may be washed away with a syringe; but I will not make any further efforts to remove it now; for I may drive it into the tympanum and kill the patient." In one case, where a foreign body had got into the ear, I extracted it, like the glass bead, with a bent probe, which I introduced very carefully behind it.

Having called your attention to this subject, I shall proceed to speak of foreign bodies in other cavities. You may find them in any cavities that have natural outlets. They may be thrust in, or they may be swallowed. They may, when swallowed, pass at once into the stomach; some, from their bulk, or irregular figure, stick in the pharynx or œsophagus; and others, even of small size, if sharp and pointed, may stick in the pharynx or tonsils.

The small bones of fish, if they be swallowed, and stick anywhere, generally do so in the tonsils. The following is not a very uncommon case:—A datient sends for you who has swallowed a fish-bone; he feels an uneasy sensation, and every time he tries to swallow he finds pain, and the more he attempts to swallow the greater is the pain. You look into his throat and see a fish-bone sticking in the tonsil. Nothing can be more easy than to hold down the tongue with one finger on the flat end of a spoon, take hold of the fish-bone with a pair of forceps and remove it. The fish-bone, however, may be stuck in the lower part of the pharynx, and then you cannot see it; but you may feel it with the finger, and having so done you may seize it with the forceps and remove it. The part at which fish-bones most frequently stick is where the œsophagus and pharynx unite just behind the cartilages of the larynx. The reason why they are so liable to stick there is, that the cartilages of the larynx are not capable of being dilated; whereas, if they pass lower down, the whole tube of the œsophagus may become dilated.

The *treatment* of these cases differs much according to circumstances—according to the exact position of the body swallowed, and according to the nature of the body itself. A person swallows a large piece of meat, and it sticks somewhere in the pharynx or œsophagus. If, on introducing the finger, you feel it quite distinctly in the pharynx, there is no reason why you should not remove it with forceps. But if it lodge in the œsophagus, then the best thing that can be done is, to introduce a common œsophageal bougie and push the piece of meat down into the stomach. A little skill is necessary in introducing the bougie. There was an Indian juggler who used to swallow a large blade sword. The sword was straight, and he pushed it readily into the stomach. The way in which it was done was this:—The man threw his head as far back as possible,—and, from early tuition, he could do that further than any one of us,—so that he made the mouth, the pharynx, and the œsophagus, one straight line, and then he introduced the sword. You should act on this principle in introducing a bougie. Let the patient be placed on a chair, as it occurs more frequently in hysterical women than others, with her head turned back as far as possible; and then having a bougie well oiled, introduce it to the pharynx, and with the finger push it down. If it meets with resistance, use moderate force to push the piece of meat into the stomach. A moderate force is always sufficient: you must be careful how you employ great force. I knew of a case where a surgeon, using a bougie roughly, pushed it through the œsophagus into the posterior mediastinum and killed the patient. I heard of another case where the same thing happened. However, it must require considerable force to push the bougie through the œsophagus; and it is only a moderate force that is necessary to push the meat into the stomach. But supposing it to be not a piece of meat, but a piece of bone, or any other foreign body; first ascertain whether it is within reach of the finger. I have already stated that a large piece of bone will generally stick in the lower part of the pharynx where that and the œsophagus unite, and you may then feel it with the finger. Endeavour to introduce the finger behind the glottis, and if you can do that seize the bone with the forceps. You must be prepared with different kinds of forceps, some of which open laterally. It may be that the foreign body lies with two flat surfaces, one to one side and the other to the other, and then the forceps that open laterally answer best. If it be in the other position, with the flat surfaces looking forwards and backwards, you must have forceps which open in another direction. You may sometimes employ shorter forceps, and in other cases longer, but they should be of tolerable length.

But let us suppose that the foreign body cannot be felt with the finger, are you then to attempt to take hold of it with forceps? Really, to extract a foreign body from the œsophagus, below the part at which you can feel it with the finger, would be a very difficult operation, and probably not a very safe one; for, in poking with the forceps you might carry them through the coats of the œsophagus. It might require great force to drive a bougie through them, but much force would not be required in order to drive through them a strong body made of steel. If the foreign body be low down, and you are to extract it at all, you must do it by other means; but probably it will be best to push it into the stomach. If it be small enough to pass the œsophagus, it certainly will be small enough to pass the pylorus; at least, in all probability. You may push it into the stomach best by means of a common bougie, or what is called a probang—a piece of whalebone with a sponge at one end. This is to be introduced into the œsophagus and pushed down towards the stomach. It may operate in two ways. It generally acts by the sponge pushing the substance into the stomach; at other times, if the foreign body do not occupy the whole diameter, but only impinges by its two shoulders, the probang may be passed below it, and as you pull up the sponge the foreign body may be drawn up with it. You make a sort of blunt hook, to be fastened to the whalebone, the intention of which is that it should be passed below the foreign body.and the foreign body dragged up by the blunt hook. The best thing, however, that you can do is to push it into the stomach, and that is the most easily acomplished.

Although it is easy to speak of dislodging these foreign bodies, you will not always find it so easy in practice; and if you cannot easily remove them, what are you to do? If the patient suffer very little inconvenience, and the part be beyond the reach of the finger, I think it is best to let them alone; but if the part be within reach of the finger, then there can be no doubt as to the propriety of attempting to remove them. If, however, there be great difficulty in dislodging the body, then it is best to let it alone, and nature will generally do what is wanted. The œsophagus will, by giving way, dilate the bowels; the fibres will contract above; and gradually the thing will creep down to the stomach; or, perhaps, it may be hawked up again. I was called to a gentleman who said that he had swallowed a large piece of fish-bone—a part of the head of a cod. I could feel nothing with the finger; I passed a bougie into the stomach, and, to state the truth, I rather doubted whether anything had lodged there. As his life was not in danger, although he was suffering some inconvenience, I thought I would let it alone. In two or three days he hawked up something, and there came away a piece of bone, larger than the thumb, which had been lodged in the œsophagus. According to my experience, in the majority of cases where foreign bodies are stuck in the œsophagus, if you fail in relieving the patient nature will accomplish it. I cannot say that I have seen any cases where any ultimate harm has arisen from a foreign body stuck in the œsophagus. Such cases have occurred, and there have been instances where a foreign body has pressed on the trachea and obstructed respiration, so that a patient has been nearly suffocated. If you are called to such a case the first thing you will do is, to make an opening into the trachea so as to enable the patient to breathe, and then you may examine the œsophagus and pharynx, and ascertain whether the foreign body can be removed or not. Cases have been recorded where an incision has been made into the œsophagus for the purpose of taking out the foreign body lodged in it; and other cases are upon record where the foreign body has occasioned suppuration of the œsophagus and an abscess in the neck, and on opening it the foreign body was found in the cavity of the abscess. Such instances, however, are very rare; and on looking over the cases recorded in the Memoirs of the French Academy of Surgery, where there is a large collection presented, drawn from authors of all ages, I do believe that, in the great majority, where the operation has been performed for the removal of foreign bodies from the œsophagus, the patients would have done much better if they had been left altogether to nature, and to the operation of their own powers.

Now, supposing the foreign body to have got into the stomach, what will it do there? Why, small bodies over and over again get into the stomach, and come away. If it be a sixpence, or a farthing, you may be pretty sure that in the course of two or three days it will be found in the evacuations. It is astonishing what foreign bodies will pass through the stomach, and go through the intestines, without doing harm. A gentleman, in a paroxysm of insanity, swallowed a pair of compasses three inches in length, and the family sent to me in great fright. The compasses had not stuck in the œsophagus, but had gone into the stomach. To think of looking for them there was quite absurd, and I told them to let him alone. He must have swallowed them with the blunt end forwards, and the probability was that they lay towards the pylorus. In the course of a fortnight, without his having suffered even a colicky pain, they one day found the compasses in his close-stool pan. He lived a considerable time afterwards, and never suffered any inconvenience from this exploit. Several persons have been in the habit of swallowing large bodies, and getting money for exhibiting the feat. A sailor, in America, in a drunken fit, swallowed a large knife. It went into the stomach, produced some colicky pains for a few days, and was then voided per anum. Two or three days afterwards he did the same thing, and finding that people stared at him, and gave him money, he went on with it. People went on purpose to see this exhibition of swallowing knives. By and by, however, he got into very ill health: there was severe colicky pain in the intestines, and in the abdomen; his stools always came away black; and he sank, and died. On examining the body several blades of knives were found, half destroyed, from the oxidation to which they had been subjected. But it seemed that the immediate cause of death was a large knife which stuck across the upper part of the rectum, running through both sides of the gut.

The great majority, even of large substances, taken into the stomach, pass through the pylorus, travel along the intestines, and find their way out at the anus. There are particular parts of the intestines, however, where these foreign bodies are most likely to stick; they may remain in the cul-de-sac of the cæcum. A woman was brought here with a tumour in the right iliac region. She died, and, on examining the body after death, an abscess was found connected with the cæcum, and in the middle of the abscess there was a pin. Over and over again women and children swallow pins, and they generally pass away without doing harm, but in this case the pin stuck in the cæcum, and getting into the cellular membrane it caused a small infiltration of feculent matter and produced the abscess. The part, however, in which foreign bodies are most likely to remain, is the rectum. No doubt that abscesses by the side of the rectum and fistulæ in ano, in many instances, arise from some foreign body sticking in the rectum. I was called to a gentleman suffering great uneasiness in the rectum. At first I thought there were piles, but when he described his symptoms more accurately I was convinced that there was something more than internal piles. I introduced my finger into the rectum, and found that there was some hard substance above the sphincter, and which appeared to be half in the gut and half out. With some difficulty I dislodged it, seized it with a pair of forceps, and removed it. It turned out to be a large core of an apple, the sharp edge having stuck in the rectum. If it had not been thus removed it would have made an abscess. I was sent for to a gentleman with a large abscess by the side of the rectum. The patient had a dry, brown tongue, and other typhoid symptoms, and I therefore concluded that it was full of putrid matter. I opened the abscess freely, and let out a large quantity of stinking putrid matter. Having done that I thought it advisable to examine the abscess with my finger, and I found a hard body, sticking in it, like a great pin. With some difficulty I removed it, and it proved to be a fish-bone, perhaps two inches in length, one end of which had stuck in the side of the rectum, and the other lay across the abscess. He had swallowed it without being aware of it; it had passed easily down the œsophagus, through the stomach and pylorus, and all the coils of the intestines and cæcum, but when it reached the rectum it passed through one side of it, allowed some of the fecal matter to intrude by its side, and cause this large abscess. Many cases are recorded by writers where the foreign bodies that have been swallowed have produced fistulæ.

When a foreign body has got into the stomach, you must consider it as out of your hands altogether, except that you must keep the bowels gently open. All violent purging should be avoided; for if there be a sharp pin, great peristaltic action may cause it to do much injury. You may exhibit lenitive electuary or castor oil, but you must not be in a hurry to expel the substance, for it will generally p ss after remaining in for a week or a fortnight, and if it be a small body it will come out much sooner. For the most part there is but little cause of apprehension, though in some cases unfortunate occurrences arise, as in the case of the woman who swallowed the pin. It is desirable to see that the substance does come away, and you must take care that the patient has his evacuations in a close-stool pan, and that they be minutely examined.

It has been proposed by the old writers to make an incision into the intestines, but at this time of day I do not think it is necessary to explain how much better it is to leave the case to nature than to have recourse to such a dangerous operation.

There is another matter of considerable practical importance to which I wish to call your attention, with respect to matters supposed to be stuck in the œsophagus. A woman was brought to town who was thought to have swallowed a piece of bone, and I believe there was no doubt that she had done so. I introduced my finger, and not being able to feel it, I concluded that it was below the reach of the finger. I then passed an œsophageal bougie into the stomach, but could not feel it; I then introduced a probang with a sponge, but with no better effect; but, still, the woman had the sensation of its being there. I now began to doubt whether it really stuck there, and to suspect that the sensation she experienced indicated that some part of the œsophagus had been abraded or torn by the foreign body, but that the body itself had passed into the stomach. It is a common trick with conjurers to put a half-crown into the hands of a person, to press it firmly, aud then to say to him, "You are sure it is there?" The party says "Yes." In fact, he has the feeling of it, but when he opens his hand it is not there. The sensation made by the pressure on the hand remains a considerable time after the body itself has been removed, especially if the feeling be assisted by the imagination. You get a piece of sand or gravel into the eye; it is taken out directly, but you persist in saying that it is there; for a little inflammation of the eye produces a feeling as if a foreign body were in the conjunctiva. So I thought it might be with this patient, who imagined that she had a bone in the œsophagus which she could not swallow. Under that impression I ordered an opiate glister; and, under its influence, the sensation was, on the next day, very much abated; and, on the following day, was entirely removed. I think that the rapid subsidence of the symptoms under this treatment proved that they depended on an injury inflicted, and not on the foreign body remaining there. I met with a similar case in the following instance.—A maid-servant was supposed to have something sticking in the œsophagus, but, with the largest bougie or probang, nothing could be discovered there. I treated her in the same manner, and, she was quite well. I suspect that this is not a very uncommon case. A person sends to you, and says that he has swallowed a fish-bone; you cannot find it; in reality, it has passed on; but it has pricked the œsophagus. By leaving such cases alone I have seen instances in which, in a day or two, the sensation has entirely disappeared.—*Lond. Lancet.*

